# Effects of Extenders Supplementation with Gum Arabic and Antioxidants on Ram Spermatozoa Quality after Cryopreservation

**DOI:** 10.3390/ani13010111

**Published:** 2022-12-28

**Authors:** Mohamed Shehab-El-Deen, Mohamed Ali, Mohammed Al-Sharari

**Affiliations:** 1 Department of Animal Production and Breeding, College of Agriculture and Veterinary Medicine, Qassim University, Buraidah 51452, Saudi Arabia; 2 Department of Animal Production, Faculty of Agriculture, Suez Canal University, Ismailia 41522, Egypt

**Keywords:** Noemi rams, semen cryopreservation, extender, gum arabic, cysteine, ascorbic acid

## Abstract

**Simple Summary:**

The current study aimed to compare the effects of the replacement of egg yolk in an extender with gum arabic along with supplementation with cysteine or ascorbic acid on semen quality and freezability in Noemi rams in vitro. Semen from six rams were collected with an artificial vagina two times per week. Spermatozoa motility and concentration were estimated with the computer-assisted semen analysis system. The semen samples were frozen using a Tris extender containing either egg yolk or gum arabic and supplemented with different concentrations of cysteine or ascorbic acid as antioxidants. The semen from each ejaculate of each ram were resuspended with a specific extender with glycerol for cryopreservation. The addition of antioxidants to both extenders has advantageous effects; however, supplementation of gum arabic extender with antioxidants cysteine (0.5 or 1 mM) or ascorbic acid (0.5 mM) has beneficial effects on semen quality after cryopreservation.

**Abstract:**

Semen cryopreservation is very important in animal agriculture to maximize the number of daughters of genetically superior males and to distribute the cryopreserved semen of good males all over the world. However, the freezing process generates some damage to sperm that reduce their fertilizing ability after thawing. Moreover, egg yolk, which is the most common animal-origin cryoprotectant used in semen dilution, is considered a source of biosecurity risk. In the current study, we aimed to compare the replacement of egg yolk in the extender by gum arabic (5%) along with supplementation with antioxidant cysteine or ascorbic acid on semen quality and freezability in Noemi rams in vitro. Semen from six rams were collected with an artificial vagina two times per week. Semen evaluation parameters such as color, volume, pH, general motility, percentage motility, concentration and cell viability ratio were assessed. Spermatozoa motility and concentration were estimated with the computer-assisted semen analysis system. The semen samples were frozen using a Tris extender containing either 15% egg yolk or 5% gum arabic. For antioxidant-supplemented extenders, cysteine or ascorbic acid was dissolved at concentrations of 0.10, 0.50 or 1.0 mM in egg yolk or gum arabic extender. The semen from each ejaculate of each ram were resuspended with a specific extender with glycerol (5%); the final volume after dilution was 1 mL semen to 4 mL extender. The samples were then cooled to 4 °C for 120 min, loaded into 0.5 mL straws and frozen in liquid nitrogen for 7 days. Supplementation of gum arabic or egg yolk extenders for ram semen with antioxidants such as cysteine or ascorbic acid has beneficial effects on semen quality after cold storage or cryopreservation. However, supplementation of a 5% gum arabic extender with cysteine at 0.5 or 1 mM concentration or ascorbic acid at 0.5 mM concentration improved the quality of spermatozoa postcryopreservation. It could be concluded that gum arabic is a good alternative for egg yolk in Noemi ram semen extenders. Antioxidants are necessary to support the addition of gum arabic to the extender to help the ram spermatozoa to survive freezing–thawing and oxidative stresses.

## 1. Introduction

Semen cryopreservation has long been used worldwide in animal agriculture to preserve genetic resources. Semen cryopreservation has participated efficiently in spreading superior genetics among countries, allowing selective breeding with desirable characteristics [1,2]. However, there are some limitations because of damage resulting from the freezing–thawing process on spermatozoa. The freezing–thawing process negatively affects membrane structure and function as well as spermatozoa survival post-thawing [3]. It is necessary to extend the semen during long storage at extremely low temperatures (−196 °C) in appropriate diluents. The Noemi breed is the most popular breed of sheep in Saudi Arabia and has a national value in local livestock production. Semen cryopreservation of Noemi sheep is important for the sustainable development of the breed. In cryopreservation, cryoprotectants such as glycerol and egg yolk are the most commonly used components in extenders. These cryoprotectants protect the spermatozoa from chilling injury during cryopreservation. However, the inclusion of egg yolk in extenders has recently become controversial because of increasing emphasis on biosecurity issues. The inclusion of egg yolk in semen extender impedes the control of pathogen transmission via semen transportation [4,5]. Furthermore, egg yolk may affect the evaluation of semen quality parameters [6]. Accordingly, finding a potential alternate for egg yolk in semen extender has become an option for researchers. The animal-free substitute effectively maintains spermatozoa viability and fertility while minimizing the risk of pathogen transmission [7,8]. Among plant-based extenders, gum arabic (GA) has been tested in stallion, ram and buck semen [9,10]. Gum arabic has shown merit to replacing egg yolk in ram semen extenders. Gum arabic may enhance the physical characteristics of semen and, hence, ram fertility. Gum arabic is a polysaccharide of natural origin obtained from the Acacia tree genus. The tree exudes a resin that contains a variable complex mixture with a high molecular weight. This exudate is made up of mainly two polysaccharide components: high-molecular-weight glycoprotein with 90% carbohydrate, and low-molecular-weight heterogeneous polysaccharide sugars [11,12]. The carbohydrate fraction of GA is galactose, rhamnose, glucuronic acid and Arabinose. It has also been suggested that GA is rich in essential and trace elements [13]. Furthermore, GA exhibits good dissolving capacity with remarkably low viscosity [14]. Accordingly, GA is a suitable substitute for macromolecules contained in egg yolk, and it can protect the spermatozoan plasma membrane from chilling injury during cryopreservation. Therefore, it would be of great importance to use a GA extender that is of nonanimal origin, well-defined and pathogen-free according to recent ram semen cryopreservation protocols.

In addition to freezing–thawing stress, cryopreserved sperm are subjected to other types of stresses including oxidative stress, which negatively affects spermatozoa quality [15,16]. Oxidative stress is a detrimental factor for semen cryopreservation due to membrane, acrosome and DNA damage of spermatozoa subjected to oxidative conditions [17]. The oxidative stress is controlled by the mitochondria, which controls the levels of reactive oxygen species (ROS) in the sperm cell. The balance between ROS formation and antioxidants in the sperm cell is crucial for capacitation, hyperactivation and acrosome reaction [18]. However, when an imbalance happens between ROS production and antioxidant levels, oxidative stress occurs; accordingly, spermatozoa quality decreases [19]. Fortunately, ROS can be balanced by supplementing the semen extender with suitable antioxidants. Ascorbic acid is proven to be a suitable antioxidant that removes ROS and stops the peroxidation process [20,21]. Likewise, cysteine is a penetrating amino acid that can enter the sperm cell and metabolize to taurine, which binds to spermatozoa membrane fatty acids and protects the cell wall against freezing-associated damages [22]. Therefore, the main goal of the present study is to compare the replacement of egg yolk in the extender with gum arabic along with supplementation of cysteine or ascorbic acid on semen quality and freezability in Noemi rams in vitro.

## 2. Materials and Methods

The current research was carried out at Agricultural and Veterinary Research Station, Sheep and Goats Unit, Qassim University, Al-Qassim region, Saudi Arabia.

### 2.1. Animals

Six healthy and fertile Noemi rams aged between 2 and 4 years were used in the current study. The rams were housed in an open shelter, provided with a clean water supply and were fed pellets and alfalfa hay. Integrated mineral licks were available as well.

### 2.2. Semen Collection

Semen was collected from each ram twice a week for 6 successive weeks by using an artificial vagina. The collected semen was immediately kept in a water bath at 37 °C. In each ejaculate, semen evaluation parameters such as color, volume, pH, general motility, percentage motility, concentration and cell viability ratio were assessed. Spermatozoa motility and concentration were estimated with the computer-assisted semen analysis system (CASA; ISAS^®^ program, Proiser R + D, Valencia, Spain). The pH was measured at room temperature according to the manufacturer’s procedure. To briefly explain, the electrode and the instrument were calibrated together before taking pH measurements. The electrode and the temperature probe were submerged approximately 4 cm into the diluter and stirred gently, and then the electrode was allowed to stabilize. The pH reading was displayed. The electrode was rinsed thoroughly with deionized water and then with some of the subsequent diluter in order to prevent cross-contamination. (HANNA HI 2211, Padova, Italy).

### 2.3. Extenders

All chemicals and media used in the current study were purchased from Sigma–Aldrich (St. Louis, MO, USA). The semen samples were frozen using a Tris extender containing either egg yolk (EY) 15% or gum arabic (GA) 5%. We have found that the 5% addition of GA gave better results in frozen semen than other concentrations when an artificial vagina used for semen collection (unpublished data). The GA was heated at 80 °C for 60 min to inactivate enzymes (modified from Ali et al. [9]). The Tris extender was prepared using 0.5 g glucose, buffering agents (3.643 g Tris and 1.7 g citric acid), 5 mL glycerol and non-pyrogenic water (added to a volume of 100 mL).

For antioxidant-supplemented extenders, cysteine was dissolved at concentrations of 0.10, 0.50 or 1.0 mM in an EY extender or a GA extender. Ascorbic acid was dissolved at concentrations of 0.10, 0.50 or 1.0 mM in an EY extender or a GA extender. The pH of the extender was adjusted using a pH meter (HANNA HI 2211, Italy) and sodium hydroxide buffer to 7.0–7.2.

### 2.4. Deep Freezing

The semen from each ejaculate of each ram were resuspended with a specific extender with glycerol (5%); the final volume after dilution was 1 mL semen to 4 mL extender, and then it was cooled to 4 °C for 120 min. After cooling, diluted semen was evaluated using the CASA system, an integrated semen analysis system (ISAS^®^ program, Proiser R + D, Paterna, Valencia, Spain), and then loaded into straws (0.5 mL). Freezing processes were performed by suspending the straws horizontally at 3–4 cm height from the surface of liquid nitrogen for 10 min to sensitize them with liquid nitrogen vapor before plunging them directly into the liquid nitrogen.

Thawing of frozen semen: at day 7 post-freezing in liquid nitrogen, straws were thawed immediately in a water bath at 37 °C for 40 s and then analyzed with the ISAS system.

### 2.5. Assessment of Spermatozoa Motility

Cooled diluted semen and frozen semen were examined for a motility pattern using the integrated semen analysis system ISAS^®^ program. A five μL sample from each diluted semen was placed in a prewarmed slide. Seven consecutive digitalized images obtained from several fields using a 10× negative-phase contrast objective (BENO phase contrast microscope, China) were examined for spermatozoa motility analysis. At least 300 spermatozoa per sample were analyzed. Subsequently, the following spermatozoa motility parameters were recorded: total motile spermatozoa (% TMS), rapid progressively motile spermatozoa (% PRS), curvilinear velocity (VCL) in μm/s, rectilinear velocity (VSL) in μm/s, the average path velocity (VAP) in μm/s, straightness index (% STR) and linearity coefficient (% LIN). Spermatozoa with a swimming speed or VAP value below 10 µm/s were considered immotile spermatozoa [9].

### 2.6. Evaluation of Cooled and Frozen Semen

After thawing, the frozen semen were evaluated for total sperm and PRS using the ISAS^®^ program, and then evaluated for plasma membrane integrity, acrosome integrity and morphological defects.

All samples of cooled diluted semen and frozen–thawed semen were evaluated for the functionality of the plasma membrane with the hypoosmotic swelling test (HOST). A solution of 100 mOsmol fructose-base was used and placed in tubes (2 mL) at 37 °C, followed by the addition of 20 μL of semen to each tube and the incubation of each tube in a water bath at 37 °C for 50 min. Subsequently, the spermatozoa were analyzed for the presence or absence of a coiled tail. One hundred spermatozoa cells were counted with phase contrast microscopy (400×) [23].

The Giemsa staining procedure was used to examine defected acrosomes [24]. At least 200 spermatozoa were examined to determine the percentages of spermatozoa with defective acrosomes. The morphologically normal spermatozoa were examined with the nigrosin–eosin stain method [25]. Acrosome defects and defective spermatozoa were determined under a light microscope (1000×).

Cell viability: fluorescent stains were used to assess cell viability. The fluorescent stain was acridine orange (AO) and propidium iodide (PI), and it was provided by Halotech DNA, S.L., Spain. Fluorescent green on the heads of spermatozoa occurs when AO is retained within intact cells. PI stain can only bind to and stain cellular DNA in damaged cells, causing them to have red fluorescence. However, PI cannot penetrate living cells. A minimum of 300 spermatozoa per sample were counted [9].

### 2.7. Statistical Analysis

Descriptive analyses were performed to evaluate variables: TMS, PRS, VCL, VSL, VAP, STR, LIN, acrosome integrity, HOST and morphological defects. One-way analysis of variance (ANOVA) was performed for statistical comparisons among groups. Analysis of the normal distribution of data was examined with the Kolmogorov–Smirnov test; if the data were not normally distributed, then a Kruskal-Wallis one-way ANOVA (non-parametric statistical test) was used to test for the presence of significant differences among all groups (SPSS, version 22). The data were considered statistically different if *p* < 0.05. Data were expressed as the means ± SEM.

## 3. Results

### 3.1. Effect of Supplemented Egg Yolk or Gum Arabic Extenders with Cysteine as Antioxidant on Ram Spermatozoa Motility and Viability in Semen Collected with Artificial Vagina

Gum arabic extender (5%) was used in conjunction with cysteine at different concentrations (0.1, 0.5 and 1 mM).

**Figure 1:**
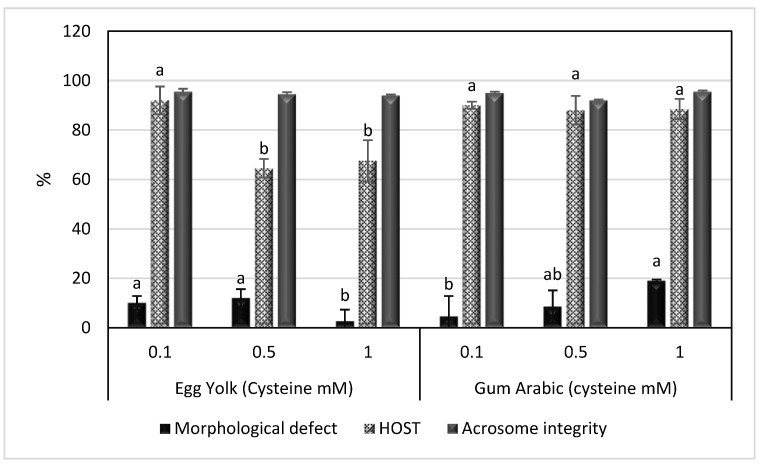
Mean ± SEM of morphological defects, HOST and acrosome integrity of spermatozoa in egg yolk or gum arabic extenders supplemented with different concentrations of cysteine (0.1, 0.5 and 1 mM) after cooling. HOST: hypoosmotic swelling test. ^a,b^ bars bearing different superscripts are significantly different (*p* < 0.05).

**Figure 2:**
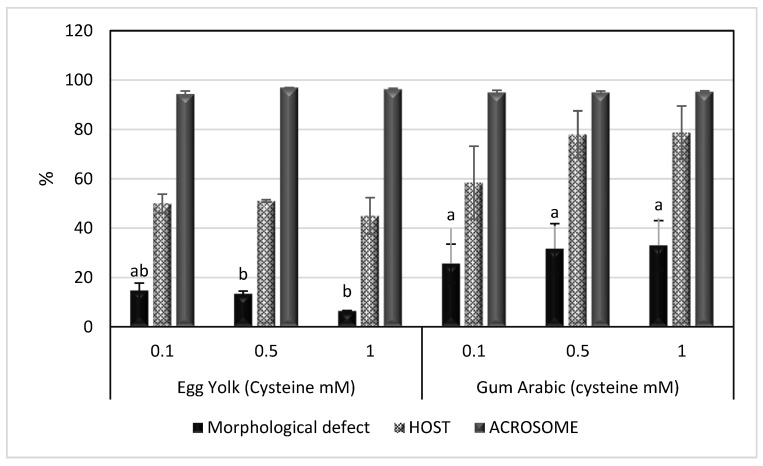
Mean ± SEM of morphological defects, HOST and acrosome integrity of spermatozoa in egg yolk or gum arabic extenders supplemented with different concentrations of cysteine (0.1, 0.5 and 1 mM) and stored by deep freezing at −196 °C. HOST: hypoosmotic swelling test. ^a,b^ bars bearing different superscripts are significantly different (*p* < 0.05).

Spermatozoa parameters, including motility, viability, plasma membrane integrity, morphological defects and acrosome integrity, were evaluated after cooling (4 °C) and freezing (−196 °C) in a Tris extender containing EY or GA with 0.1, 0.5 and 1 mM cysteine. The results are presented in Table 1 and Table 2 and Figure 1 and Figure 2. There were no significant effects of cysteine supplementation on the TMS, PRS, VCL, VSL, VAP or LIN of spermatozoa in either EY or GA extenders. However, the morphological defects were significantly lower in EY extenders supplemented with 1 mM cysteine and GA extenders supplemented with 0.1 mM cysteine (*p* < 0.05), as shown in Figure 1. The HOST was higher in EY extenders supplemented with 0.1 mM cysteine and GA extenders supplemented with any studied cysteine concentration. However, no significant differences could be found in acrosome integrity among groups, as also shown in Figure 1.

After deep freezing, GA extenders supplemented with 0.5 or 1 mM cysteine improved the TMS, PRS, VCL, VSL and VAP of spermatozoa post-freezing, as shown in Table 2 (*p* < 0.05). However, the morphological defect rate was significantly higher in these extenders; GA extenders supplemented with 0.5 or 1 mM cysteine compared to other groups. There were no significant differences in HOST and acrosome integrity among groups, as also shown in Figure 2.

### 3.2. Effect of Supplemented Egg Yolk or Gum Arabic Extenders with Ascorbic Acid as Antioxidant on Ram Spermatozoa Motility and Viability in Semen Collected with Artificial Vagina

**Figure 3:**
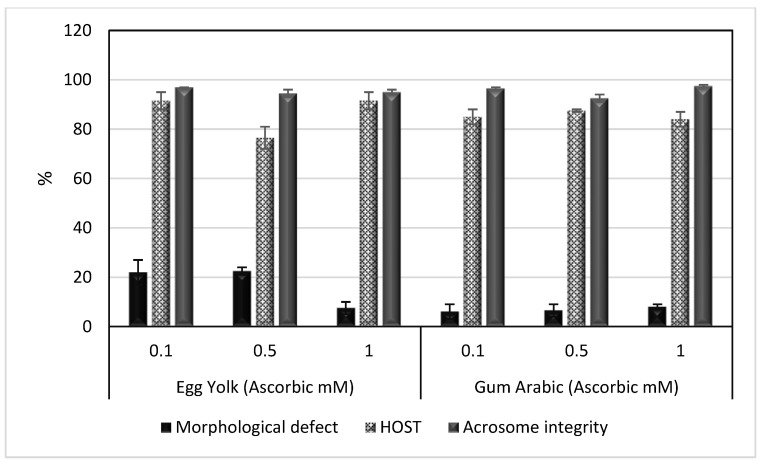
Mean ± SEM of morphological defects, HOST and acrosome integrity of spermatozoa in egg yolk or gum arabic extenders supplemented with different concentrations of ascorbic acid (0.1, 0.5 and 1 mM) after cooling. HOST: hypoosmotic swelling test.

**Figure 4:**
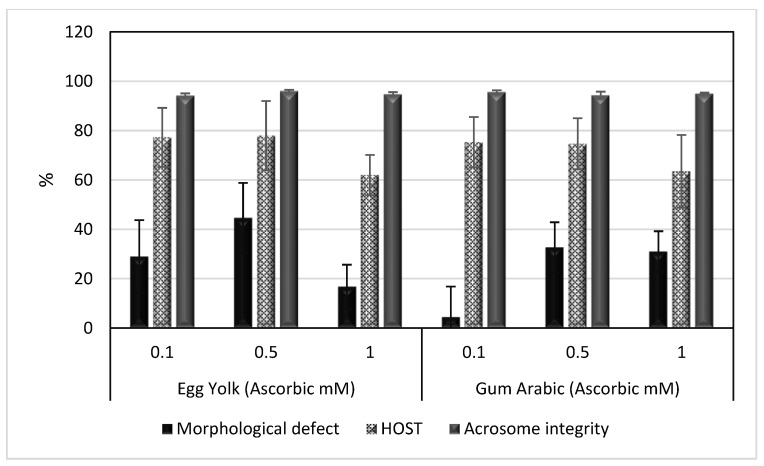
Mean ± SEM of morphological defects, HOST and acrosome integrity of spermatozoa in egg yolk or gum arabic extenders supplemented with different concentrations of ascorbic acid (0.1, 0.5 and 1 mM) and stored by deep freezing at −196 °C. HOST: hypoosmotic swelling test.

The results of this experiment are summarized in Table 3 and Table 4 and Figure 3 and Figure 4. It was obvious from the results that GA extenders supplemented with 1 mM ascorbic acid significantly improved the TMS, PRS, VCL, VSL and VAP of spermatozoa after cold storage (*p* < 0.05), as shown in Table 3. However, post-freezing results showed that GA extenders supplemented with 0.5 mM ascorbic acid gave better results in TMS, PRS, VCL, VSL, VAP and LIN of spermatozoa (*p* < 0.05), as shown in Table 4. There were no significant differences in morphological defects, HOST or acrosome integrity among groups after cold storage or freezing, as shown in Figure 3 and Figure 4.

**Figure 5:**
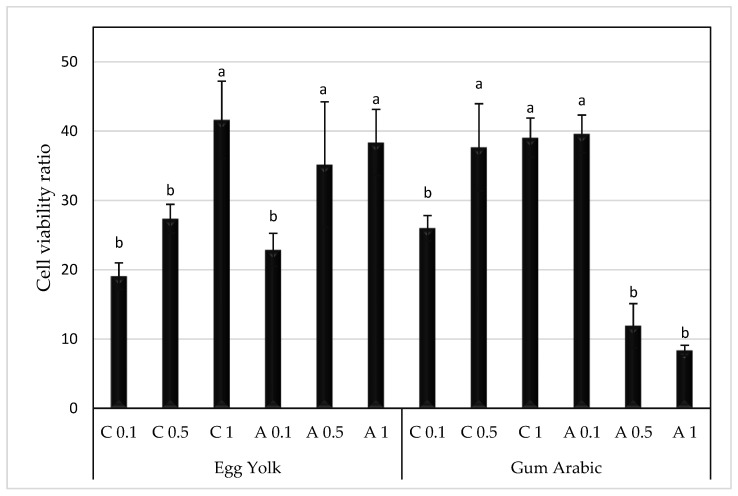
Mean ± SEM of cell viability after adding cysteine or ascorbic acid to egg yolk or gum arabic extenders for ram spermatozoa postcryopreservation. C: cyctein (mM); A: ascorbic acid (mM). ^a,b^ bars bearing different superscript letters are significantly different from one another (*p* < 0.05).

The results of the extenders’ supplementation with cysteine or ascorbic acid on sperm cell viability ratio post thawing are represented in Figure 5.

Cell viability ratio was significantly enhanced by a supplementation of 1 mM cysteine to an EY extender or 0.5–1 mM to a GA extender (*p* < 0.05). Similarly, ascorbic acid increased sperm cell viability when added to an EY extender at concentrations of 0.5–1 mM (*p* < 0.05). However, ascorbic acid gave better results of cell viability when added to a GA extender at the lowest concentration (0.1 mM) (*p* < 0.05).

## 4. Discussion

In the current study, gum arabic extenders combined with antioxidants were used for Noemi ram semen cryopreservation. Extenders are the most important part of the semen cryopreservation process because they provide spermatozoa the capability of maintaining motility and viability outside of the reproductive tract and for a long period during storage at extremely low temperatures. Cryoprotectants are components that are used in extenders to provide protection to spermatozoa from cryogenic stress [26,27]. In addition to the biosecurity risks of EY inclusion in extenders, there is evidence that EY reduces the motility of bull sperm by the granules found in EY [28]. Moreover, EY supplementation has an issue regarding the accuracy of computer-assisted analysis: false spermatozoa detection that might be caused by EY particles or accompanying debris [29]. This could ultimately lead to elevating the immotile sperm percentage.

The results of the current experiment indicated that maintaining the motility of the sperm, especially progressively motile sperm could be attained more satisfactorily in extenders supplemented with AG than those supplemented with egg yolk for ram semen. These results are in agreement with another study that added AG to the cryopreservation of horse semen [9]. Gum arabic is complex, comprised of polysaccharides [30,31] and superior to penetrating cryoprotectants such as glycerol, DMSO or lactose [26]. Gum arabic solubility in water is good in relatively high concentrations. The mode of protective action of polysaccharides is linked to their control of amorphous matrices adjacent to ice crystals, which enhances the stability of frozen solutions around the spermatozoa [32]. The AG has a wide variety of polysaccharides that can support the extender and protect the exogenous microenvironment of spermatozoa.

The progressively motile spermatozoa in the AG extenders post-thawing were attributed to the AG’s effects on solution homogeneity of the diluter in terms of pH, viscosity and osmolality. Additionally, this phenomenon gave better opportunity for sperm survival post-thawing [9]. However, changes in membrane permeability, spermatozoa functionality and metabolism could lead to drastic changes in sperm motility and fertilizability [33]. However, the exact mode of protective action caused by AG is still not fully understood. Therefore, more efforts are needed to uncover the important protective role of AG on sperm during cryopreservation. A different suggestion for spermatozoa survival in AG diluent might be related to energy molecules and ATP concentration during storage. Our results suggested AG at a good concentration provides suitable velocity (VCL, VSL and VAP). Apparently, higher concentrations of more than five percent could affect the resistance of solution to fast-swimming spermatozoa due to increased velocity. It has been suggested that the diluent viscosity can hamper the motility pattern of spermatozoa [9,34,35]. Other studies reported that various viscosity degrees could be obtained by the addition of Ficoll, carboxy methyl cellulose, methylcellulose or egg yolk.

Freezing–thawing stress during the cryopreservation process is indicated to be responsible for drastic irreversible cellular damage, which ultimately leads to inferior semen fertility. However, the differences in morphological defects post-freezing among diluents were insignificant. The spermatozoa plasma membrane permits crucial molecules to transfer through the cytoplasm. Moreover, integrity of sperm plasma membrane is very crucial for the integration during fertilization process between the male and female gametes [36,37]. The superiority of AG and EY as demonstrated with HOST and biometric testing suggested that it might provide better protection for the plasma membrane during deep freezing.

Antioxidants such as cysteine and ascorbic acid were added into a freezing extender that was used for semen cryopreservation and oocyte and embryo preservation [38]. However, the imbalance between ROS and the antioxidant, or the inability of the antioxidant to tolerate the ROS during semen cryopreservation, will damage the spermatozoa due to oxidative stress that occurs during semen cryopreservation [39,40]. The lipid peroxidation of ram spermatozoa increases during semen preservation procedures, especially in the spermatozoa midpiece.

Supplementing post-thawed semen with cysteine or ascorbic acid had a positive effect on spermatozoa motility and spermatozoa membrane integrity and viability compared to controls. This result was similar to the research of Sariözkan et al. [41], who reported that the addition of cysteine exhibited significant protection on post-thawed bull semen motility, viability and membrane integrity. However, the results of the present study are not in agreement with other studies conducted on rams [42], goats [43] and horses [44], as these other studies reported a high correlation between ROS and spermatozoa motility and spermatozoa membrane integrity and viability [45]. This effect may be due to the lipid peroxidation of the spermatozoa membrane that promotes axonemal protein phosphorylation. However, the study by Alamaary et al. [44] showed that low concentration of cysteine and ascorbic acid shows better motion parameters than those with higher concentration. This means that spermatozoa in an extender with a low concentration of cysteine and ascorbic acid are protected from oxidative stress.

The spermatozoa motility patterns (VSL, VCL, VAP, STR and LIN) are the best indicators of spermatozoa capacity and hyperactivity to predict the spermatozoa’s ability to penetrate the oocytes’ zona pellucida [46]. However, high spermatozoa velocity is too closely associated with the total percentage of spermatozoa’s progressive motility to consider as a critical criterion [47]. The additions of cysteine and ascorbic acid in the present results have an effective effect on spermatozoa velocity. However, the compositions of extenders containing AG with cysteine have a strong influence on the quality of semen after freezing. These results correspond well with a previous study [48]: stallion semen cryopreserved with cysteine had low levels of oxidative stress in all studied concentrations, while the semen frozen with ascorbic acid had greater oxidative stress compared to the control group. However, this finding is not in agreement with the previous studies of Çoyan et al. [49] that reported no effect of the addition of cysteine in preventing lipid peroxidation in the cryopreserved semen of rams. This is inconsistent with past findings. For instance, studies using bovine [50] and goats [43] found that freezing extenders that were supplemented with ascorbic acid protected the post-thawed semen from oxidative stress. In addition, a study on dogs reported a negative influence of canine spermatozoa quality parameters after adding different concentrations of ascorbic acid [51].

We hypothesized that the acidification of the post-thawed semen with ascorbic acid during the freezing procedure would increase the oxidative stress in the samples. A prior study [52] noted a pH decline during long-term storage. Furthermore, pH highly affects ROS as suggested in previous research [53]. Cysteine and ascorbic acid play dual roles in ROS. Despite their ability to scavenge ROS, they can be harmful to spermatozoa. However, ascorbic acid can exhibit oxidizing activity in the presence of transition metals [54]. In agreement with our findings, Michael et al. [51] reported an increase in free radical production in cryopreserved semen that were treated with ascorbic acid. These results could be due to the change in the pH in the post-thawed semen treated with ascorbic acid.

However, the additions of antioxidant cysteine or ascorbic acid in the current study did not show a capacity to protect acrosome integrity and plasma membrane integrity during cryopreservation when compared to the control. The spermatozoa morphology in these concentrations provides poor results in extenders (EY and AG) with cysteine and ascorbic acid compared to the control group. The same result was observed in a study by Ceylan et al. [55] that used cooled dog semen. However, normal spermatozoa morphology and the acrosome structure are insufficient to indicate the spermatozoa’s acrosome function.

## 5. Conclusions

Gum arabic could be used as a substitute for egg yolk in ram semen extenders without apparent negative effects. Supplementation of gum arabic or egg yolk extenders for ram semen with antioxidants such as cysteine or ascorbic acid has beneficial effects on semen quality after cold storage or cryopreservation. However, a 5% gum arabic extender gave better results after cryopreservation when supplemented with 0.5 or 1 mM of cysteine or with ascorbic acid at a concentration of 0.5 mM.

Ultimately, gum arabic can be recommended as a good alternative for egg yolk in Noemi ram semen extenders. Antioxidants are necessary to support the addition of gum arabic to extenders to help the ram spermatozoa survive freezing–thawing and oxidative stresses. Our findings can help cryopreserve and spread genetically superior Noemi ram semen efficiently.

## Figures and Tables

**Table 1:** Spermatozoa motility and viability parameters (mean ± SEM) in egg yolk and gum arabic extenders supplemented with different concentrations of cysteine (0.1, 0.5 and 1 mM) after cooling.

	Egg Yolk (15%) (Cysteine mM)			Gum Arabic (5%) (Cysteine mM)		
	0.1	0.5	1	0.1	0.5	1
TMS	96.25 ± 0.25	98.55 ± 0.05	87.40± 2.1	95.65 ± 0.55	97.10 ± 0.2	97.40 ± 0.7
PRS	78.20 ± 0.98	89.15 ± 0.25	86.90 ± 2.6	89.20 ± 2.3	84.95 ± 2.85	85.65 ± 4.45
VCL	99.95 ± 0.45	115.8 ± 0.15	110.75 ± 2.4	118.15 ± 1.35	108.9 ± 3.0	110.10 ± 5.2
VSL	28.10 ± 0.1	29.65 ± 0.15	26.50 ± 5.0	30.80 ± 0.0	28.70 ± 1.2	33.55 ± 1.1
VAP	54.25 ± 0.05	61.05 ± 0.35	53.50 ± 1.8	61.95 ± 0.45	57.80 ± 1.8	58.40 ± 2.9
LIN	28.15 ± 0.05	25.60 ± 0.0	26.60 ± 1.06	26.10 ± 0.3	26.30 ± 0.4	26.40 ± 0.4
STR	51.85 ± 0.05 ^b^	48.60 ± 0.0 ^b^	49.55 ± 1.05 ^b^	49.75 ± 0.3 ^b^	49.6 ± 0.5 ^b^	79.75 ± 0.65 ^a^

TMS: Total motile spermatozoa (%); PRS: Rapid progressively motile spermatozoa (%); VCL: Curvilinear velocity (μm/s); VSL: rectilinear velocity (μm/s); VAP: average path velocity (μm/s); LIN: linearity coefficient (%); STR: straightness index (%); ^a,b^ means in the same row with different superscript letters are significantly different from one another (*p* < 0.05).

**Table 2:** Spermatozoa motility and viability parameters (mean ± SEM) in egg yolk and gum arabic extenders supplemented with different concentrations of cysteine (0.1, 0.5 and 1 mM) and stored by deep freezing at −196 °C.

	Egg Yolk (15%) (Cysteine mM)			Gum Arabic (5%) (Cysteine mM)		
	0.1	0.5	1	0.1	0.5	1
TMS	10.03 ± 0.5 ^b^	27.5 ± 0.4 ^b^	16.56 ± 3.1 ^b^	32.04 ± 13.3 ^b^	79.9 ± 0.7 ^a^	69.43 ± 0.8 ^a^
PRS	3.06 ± 0.1 ^b^	11.33 ± 0.3 ^b^	6.03 ± 1.9 ^b^	18.28 ± 9.06 ^b^	58.26 ± 0.6 ^a^	47.46 ± 1.4 ^a^
VCL	48.06 ± 0.4 ^b^	58.13 ± 0.3 ^b^	47.66 ± 4.3 ^b^	53.32 ± 3.1 ^b^	70.7 ± 1.4 ^a^	65.46 ± 1.3 ^a^
VSL	14.36 ± 0.1 ^b^	17.53 ± 0.1 ^ab^	14.86 ± 0.5 ^b^	16.14 ± 1.3 ^ab^	20.56 ± 0.21 ^a^	19.30 ± 0.5 ^a^
VAP	26.46 ± 0.1 ^b^	32.30 ± 0.2 ^b^	27.06 ± 1.3 ^b^	30.16 ± 2.2 ^b^	39.63 ± 0.6 ^a^	37.0 ± 0.6 ^a^
LIN	29.83 ± 0.46	30.20 ± 0.05	31.5 ± 1.65	30.12 ± 0.73	29.06 ± 0.29	29.40 ± 0.17
STR	54.20 ± 0.4	54.40 ± 0.1	54.93 ± 0.8	53.40 ± 0.3	51.90 ± 0.3	52.13 ± 0.4

TMS: Total motile spermatozoa (%); PRS: Rapid progressively motile spermatozoa (%); VCL: Curvilinear velocity (μm/s); VSL: rectilinear velocity (μm/s); VAP: average path velocity (μm/s); LIN: linearity coefficient (%); STR: straightness index (%); ^a,b^ means in the same row with different superscript letters are significantly different from one another (*p* < 0.05).

**Table 3:** Spermatozoa motility and viability parameters (mean ± SEM) in egg yolk and gum arabic extenders supplemented with different concentrations of ascorbic acid (0.1, 0.5 and 1 mM) after cooling.

	Egg Yolk (15%) (Ascorbic Acid mM)			Gum Arabic (5%) (Ascorbic Acid mM)		
	0.1	0.5	1	0.1	0.5	1
TMS	93.65 ± 0.2 ^b^	97.55 ± 0.05 ^a^	93.0 ± 0.5 ^b^	78.65 ± 0.2 ^b^	91.20 ± 0.4 ^b^	98.70 ± 0.1 ^a^
PRS	76.35 ± 0.4 ^b^	78.7 ± 0.1 ^b^	68.60 ± 0.5 ^b^	50.10 ± 0.3 ^b^	77.20 ± 0.8 ^b^	95.55 ± 0.3 ^a^
VCL	94.0 ± 0.8 ^b^	101.85 ± 0.05 ^b^	89.55 ± 0.2 ^b^	79.55 ± 0.05 ^b^	109.80 ± 1.0 ^a^	132.20 ± 0.6 ^a^
VSL	30.25 ± 0.2 ^b^	26.80 ± 0.01 ^b^	26.50 ± 0.2 ^b^	23.35 ± 0.1 ^b^	27.0 ± 0.2 ^b^	32.10 ± 0.2 ^a^
VAP	53.50 ± 0.2 ^b^	55.65 ± 0.05 ^ab^	49.70 ± 0.1 ^b^	42.05 ± 0.05 ^b^	56.25 ± 0.4 ^a^	67.45 ± 0.3 ^a^
LIN	32.20 ± 0.6 ^a^	26.35 ± 0.05 ^b^	29.60 ± 0.3 ^b^	29.35 ± 0.1 ^b^	24.60 ± 0.0 ^b^	24.30 ± 0.01 ^b^
STR	56.60 ± 0.7 ^a^	48.45 ± 0.2 ^b^	35.30 ± 0.5 ^b^	55.60 ± 0.2 ^a^	48.0 ± 0.01 ^b^	47.60 ± 0.0 ^b^

TMS: Total motile spermatozoa (%); PRS: Rapid progressively motile spermatozoa (%); VCL: Curvilinear velocity (μm/s); VSL: rectilinear velocity (μm/s); VAP: average path velocity (μm/s); LIN: linearity coefficient (%); STR: straightness index (%); ^a,b^ means in the same row with different superscript letters are significantly different from one another (*p* < 0.05).

**Table 4:** Spermatozoa motility and viability parameters (mean ± SEM) in egg yolk and gum arabic extenders supplemented with different concentrations of ascorbic acid (0.1, 0.5 and 1 mM) and stored by deep freezing at −196 °C.

	Egg Yolk (15%) (Ascorbic Acid mM)			Gum Arabic (5%) (Ascorbic Acid mM)		
	0.1	0.5	1	0.1	0.5	1
TMS	26.05 ± 2.0 ^b^	28.00 ± 0.1 ^b^	28.12 ± 7.5 ^b^	31.06 ± 12.8 ^b^	42.10 ± 17.65 ^a^	17.70 ± 1.37 ^b^
PRS	8.62 ± 0.6 ^b^	10.40 ± 0.1 ^b^	10.02 ± 3.0 ^b^	15.73 ± 7.1 ^b^	40.96 ± 1.3 ^a^	5.87 ± 0.5 ^b^
VCL	49.20 ± 0.6 ^b^	51.73 ± 0.1 ^b^	53.12 ± 2.7 ^b^	51.73 ± 2.4 ^b^	64.73 ± 0.2 ^a^	50.80 ± 0.9 ^b^
VSL	15.75 ± 0.2 ^b^	18.06 ± 0.03 ^a^	15.52 ± 0.8 ^b^	15.43 ± 1.1 ^b^	19.26 ± 0.06 ^a^	15.02 ± 0.2 ^b^
VAP	28.62 ± 0.5 ^b^	30.46 ± 0.03 ^b^	29.07 ± 1.6 ^b^	28.50 ± 1.8 ^b^	35.40 ± 0.1 ^a^	28.40 ± 0.5 ^b^
LIN	31.97 ± 0.09 ^a^	34.96 ± 0.1 ^a^	30.55 ± 0.2 ^a^	20.73 ± 8.6 ^b^	29.73 ± 0.03 ^a^	29.57 ± 0.2 ^a^
STR	54.97 ± 0.2 ^b^	59.23 ± 0.08 ^a^	53.37 ± 0.1 ^b^	54.0 ± 0.4 ^b^	54.40 ± 0.05 ^b^	52.90 ± 0.2 ^b^

TMS: Total motile spermatozoa (%); PRS: Rapid progressively motile spermatozoa (%); VCL: Curvilinear velocity (μm/s); VSL: rectilinear velocity (μm/s); VAP: average path velocity (μm/s); LIN: linearity coefficient (%); STR: straightness index (%); ^a,b^ means in the same row with different superscript letters are significantly different from one another (*p* < 0.05).

## Data Availability

The data presented in this study are available on request from the corresponding author.
